# National-scale 10-m maps of cropland use intensity in China during 2018–2023

**DOI:** 10.1038/s41597-024-03456-0

**Published:** 2024-06-26

**Authors:** Bingwen Qiu, Baoli Liu, Zhenghong Tang, Jinwei Dong, Weiming Xu, Juanzhu Liang, Nan Chen, Jiangping Chen, Laigang Wang, Chengming Zhang, Zhengrong Li, Fangzheng Wu

**Affiliations:** 1 Key Laboratory of Spatial Data Mining &Information Sharing of Ministry of Education, Academy of Digital China (Fujian), Fuzhou University, Fuzhou, 350116 Fujian China; 2 Community and Regional Planning Program, University of Nebraska-Lincoln, Lincoln, 68558 Nebraska USA; 3 Institute of Geographic Sciences and Natural Resources Research, Chinese Academy of Sciences, Beijing, 100101 China; 4 School of Remote Sensing and Information Engineering, Wuhan University, Wuhan, China; 5 Institution of Agricultural Economy and Information, Henan Academy of Agricultural Sciences, Zhengzhou, China; 6 College of Information Science and Engineering, Shandong Agricultural University, Taian, China

**Keywords:** Agriculture, Geography

## Abstract

The amount of actively cultivated land in China is increasingly threatened by rapid urbanization and rural population aging. Quantifying the extent and changes of active cropland and cropping intensity is crucial to global food security. However, national-scale datasets for smallholder agriculture are limited in spatiotemporal continuity, resolution, and precision. In this paper, we present updated annual Cropland Use Intensity maps in China (China-CUI10m) with descriptions of the extent of fallow/abandoned, actively cropped fields and cropping intensity at a 10-m resolution in recent six years (2018–2023). The dataset is produced by robust algorithms with no requirements for regional adjustments or intensive training samples, which take full advantage of the Sentinel-1 (S1) SAR and Sentinel-2 (S2) MSI time series. The China-CUI10m maps have achieved high accuracy when compared to ground truth data (Overall accuracy = 90.88%) and statistical data (R^2^ > 0.94). This paper provides the recent trends in cropland abandonment and agricultural intensification in China, which contributes to facilitating geographic-targeted cropland use control policies towards sustainable intensification of smallholder agricultural systems in developing countries.

## Background and summary

The Sustainable Development Goals (SDGs) aim to achieve zero hunger by 2030^1^. More foods are expected to be produced to ensure global food security^2^. However, global food security is being constrained by a series of issues such as the cropland loss from urbanization, the widespread cropland abandonment, and the aging agricultural population in smallholder farming systems^3^. Increasing food production is further challenged by uncertainties introduced by regional conflicts and climate changes^4,5^. Big earth data boosts United Nations (UN) SDGs^6^. Accurate and updated geospatial data on cropland is critical for grain production management^7^. Sustainable intensification can enhance crop production on existing cropland with no demand for cropland expansion^8^. Spatiotemporal explicit information on cropland use intensity is required to gain knowledge on the capability for agricultural intensification^9^. However, there is a gap in data availability and technologies^10^.

Spatiotemporal continuous Cropland Use Intensity (CUI) maps provide updated descriptions of active cropland and cropping intensity. CUI maps are the foundations for agricultural applications such as accurately identifying crop types and estimating crop yields, which is crucial to global food security^11^. Existing land use/cover datasets provided information on cropland distribution, which cannot reflect the highly dynamic agricultural land use intensity over regions and years^9^. There were large inconsistencies and uncertainties regarding the cropland data in existing land cover products due to the complexity of cropland, which can be actively cropped by different cropping intensities (single or multiple cropping) or even left fallowed^7,12,13^. Despite considerable studies on cropland mapping, little attention has been directed to identifying actively cropped fields from fallowed/abandoned cropland at large scales^12^. Large-scale LUI data products are limited in medium or coarse resolutions, one single year, and classification accuracies, especially in smallholder agricultural systems^9,11,14^.

China has the largest multiple cropping systems dominated by small-holder farms in the world^15^. China ranks first in global cereal production^16^. The cropland and cropping systems have significantly changed due to combined influences from a series of agricultural policies targeting ensuring food self-sufficiency, continuous urbanization, and rural revitalization^17,18^. Historical investigations on 500-m MODIS estimated land cover changes revealed substantial cropland abandonments during the past few decades (1990–2019)^19^. Agricultural land use intensity experienced significant changes during the early 21^st^ century (2000–2015) according to the MODIS Vegetation Indices (VI) derived cropping intensity datasets^20^. These MODIS-based data products confronted challenges in depicting the small-holder farm in China, due to the mixed-pixel problems of MODIS data and the low data availabilities of optical images in cloudy regions^21^. Spatiotemporal continuous data updated products on cropland use intensity with finer resolutions are still unavailable in recent years in China, which hinders our understanding of changes in agricultural intensification and future directions^22^.

There are great opportunities for generating large-scale datasets on agricultural systems with finer resolutions with the increasing availability of time series images such as the Sentinel-1 (S1) SAR and Sentinel-2 (S2) MSI datasets^23,24^. Land use mapping accuracy could be improved by incorporating spectral indices based on red-edge bands^25^ and fusing optical and SAR datasets^26,27^. Recent progress has been made in generating 10–30 m national-scale datasets on cropping intensity and crop types over conterminous China^11,21,28^. However, these related studies were limited in one specific year and lack of recent updated data products since 2020. Additionally, these few recent studies on national-scale 10-m CI mapping applied the 30-m cropland data products developed based on Landsat images^22^. There were two recently published 10-m global cropland datasets, the ESA_WorldCover and the FROM_GLC^29^. However, these recent 10-m cropland datasets remain underexploited in the fields of agricultural mapping in China. This study aimed to generate spatiotemporal continuous national-scale 10-m maps of cropland use intensity in China during the period 2018–2023. All available S1 and S2 time series datasets were used for agricultural mapping based on a robust approach through combined considerations of vegetative and productive Stages (MILS)^28^. Our consistent datasets on cropland-use intensity can be applied to measure the extent and dynamics of cropland abandonment and actively cropped fields as well as cropping frequency in China.

## Methods

### Study area

China is characterized by frequently varying complex cropping systems across a wide range of climatic and topographic conditions. The cropland spread from the tropical, subtropical zone in southern China to the cold temperature in northern China. A large majority of cropland is quantified for multiple cropping concerning the climatic conditions^30^, especially the southern and central China. China is dominated by smallholder farms with diverse cropping patterns^31^. Dominated multiple cropping patterns included winter wheat plus maize/rice/peanut/soybean, oilseed/tobacco/vegetable-rice, and double rice^32,33^. These three staple crops (maize, rice, and wheat) are widely distributed in China, which accounted for more than half (57.08%) of the total sown area in China in 2020 (http://www.stats.gov.cn/english/). According to the agricultural census data, there were 1,278,618 Km^2^ (1.918 billion mu) of cropland in China in 2019. It is unknown whether the actively cropped area has reduced or not in recent years since there have been no updated officially reported data on cropland since 2019.

### Sentinel-1 SAR time series images

We used both the SAR and optical time-series images. The SAR images applied were the Interferometric Wide Swath (IW) instrument mode with dual-band cross-polarization (VV) and horizontal receive (VH). All available level-1 Ground Range Detected (GRD) data provided by the Google Earth Engine platform were utilized, undergoing processing for thermal noise removal, radiometric calibration, and terrain correction (https://developers.google.com/earth-engine/guides/sentinel1). A smoothed 12-day composite time series was generated for the VH and VV backscattering coefficient datasets using the Whittaker Smoother (WS) (with parameters set as lambda = 1 and order = 2) for each year^34^. The Sentinel-1 SAR time series data were processed in the Google Earth Engine platform.

### Multiple spectral indices time series based on Sentinel-2 MSI images

We used all available Sentinel-2A/B MultiSpectral Instrument (MSI) images in China during the study period of 2018–2023. We discarded invalid observations with cloud contamination or cloud shallow concerning the quality band (QA60). Four kinds of spectral indices were computed to characterize cropland from multiple dimensions. The first kind of spectral index was the commonly applied Vegetation Index (VI), the two-band Enhanced Vegetation Index (EVI2)^35^. The EVI2 ranges within [−1,1] and higher values represented a higher density of vegetation cover. The second kind of spectral index was the soil index, Dry Bare-Soil Index (DBSI)^36^. DBSI values range within [−2,2], where larger values reveal higher soil bareness^36^. The third spectral index was the pigment index, the Chlorophyll (Chl) Index red edge (CIre)^37^. The CIre values are above zero, where larger values suggest stronger chlorophyll concentration^37^. The fourth spectral index was the pigment index, the Browning Reflectance Index (BRI)^38^. The BRI index generally ranges within [−6,14], where large BIR values indicate high carotenoid/chlorophyll ratios. These four spectral indices were calculated using the following functions (functions 1–4).

1 $$EVI2=2.5\times ({\rho }_{NIR}-{\rho }_{{\rm{R}}{\rm{e}}{\rm{d}}})/({\rho }_{NIR}+2.4\times {\rho }_{{\rm{R}}{\rm{e}}{\rm{d}}}+1)$$

2 $$DBSI=\frac{{\rho }_{SWIR}-{\rho }_{Green}}{{\rho }_{SWIR}+{\rho }_{Green}}-\frac{{\rho }_{NIR}-{\rho }_{{\rm{R}}{\rm{e}}{\rm{d}}}}{{\rho }_{NIR}+{\rho }_{{\rm{R}}{\rm{e}}{\rm{d}}}}$$

3 $$CIre=\frac{{\rho }_{754}}{{\rho }_{709}}-1=\frac{{\rho }_{VRE1}}{{\rho }_{VRE3}}-1$$

4 $$BRI=\frac{1/{\rho }_{Green}-1/{\rho }_{VRE1}}{{\rho }_{NIR}}$$

Where ${\rho }_{NIR}$, ${\rho }_{VRE3}$ and ${\rho }_{SWIR}$ represented the TOA values from the Near-infrared, Red, Green, Vegetation Red Edge1 (VRE1), Vegetation Red Edge3 (VRE3), and Short-Wave Infrared Red band (SWIR) in Sentinel-2 images, respectively.

The 10-day composite smoother time series were developed for each spectral index on individual year according to the following three steps: (1) 10-day composites were produced using the medium values calculated based on valid S2 observations; (2) data gaps were filled by linear interpolation to achieve spatiotemporal continuity; (3) the 10-day time series datasets was smoothed with the Whittaker Smoother (WS) (lambda = 10, order = 2)^34^. The WS smoother demonstrated good performances in multiple cropping regions and therefore was applied here^39^.

**Fig. 1:**
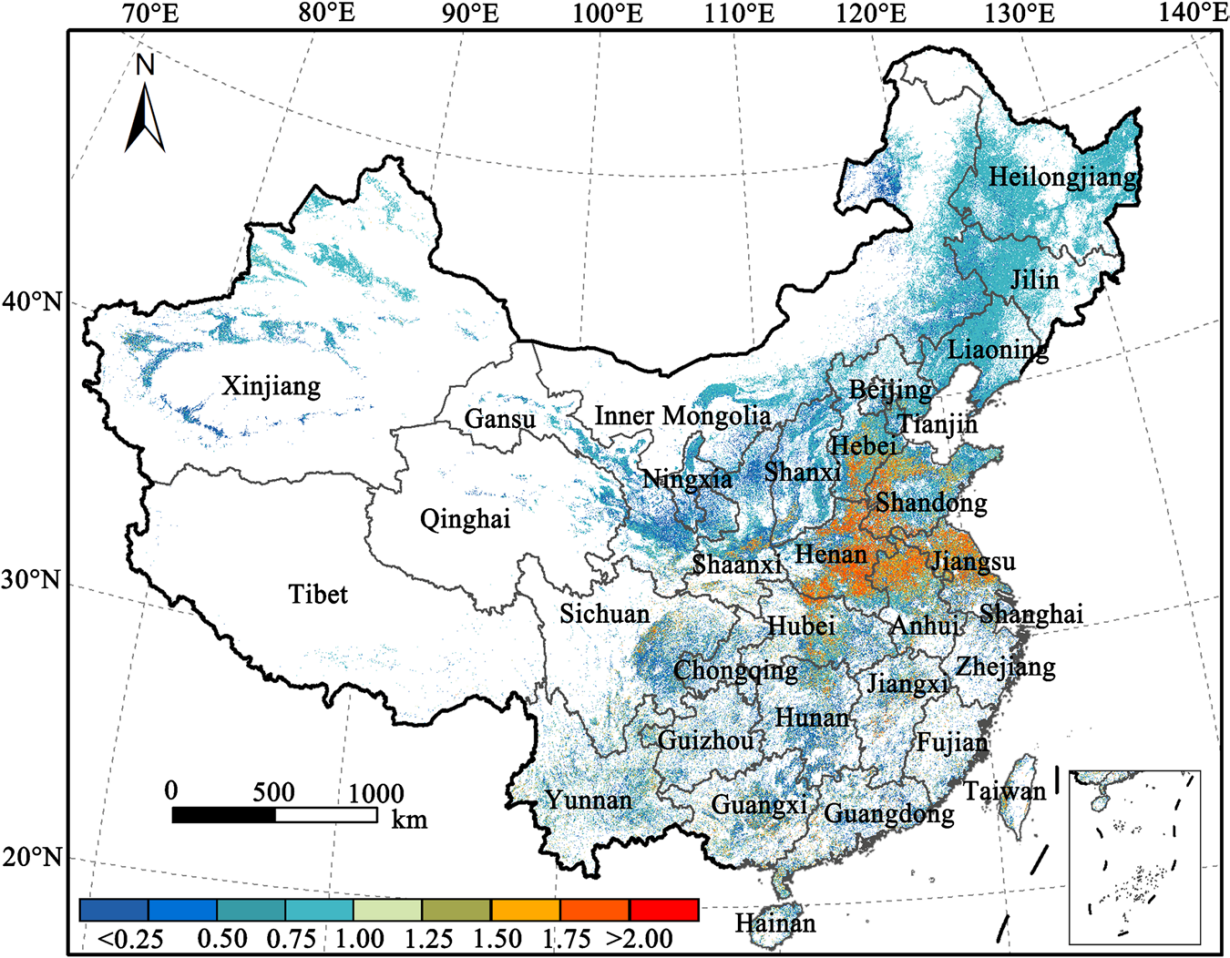
The map of averaged cropland use intensity in China.

The vegetation indices of NDVI or EVI2 have been widely applied to extract phenological metrics for mapping cropland, cropping intensity, and crop types^11,21,40^. The start and end of the crop growing season were characterized by bare soil^41^, and therefore the temporal profiles of vegetation and soil indices can be exploited to describe the cropping activities in cropland. Cropland fully covered by crops during heading stages illustrates high vegetation density (high EVI2), low soil bareness (low DBSI), and strong chlorophyll concentration (high CIre). These red-edge-based spectral indices are sensitive to the changes in canopy pigment concentration, which proved to be useful in agricultural remote sensing applications such as estimating crop phenology and crop types^42,43^. However, the performances on measuring LUI remain under-exploited Fig. 1.

### Validation data and other datasets

**Fig. 2:**
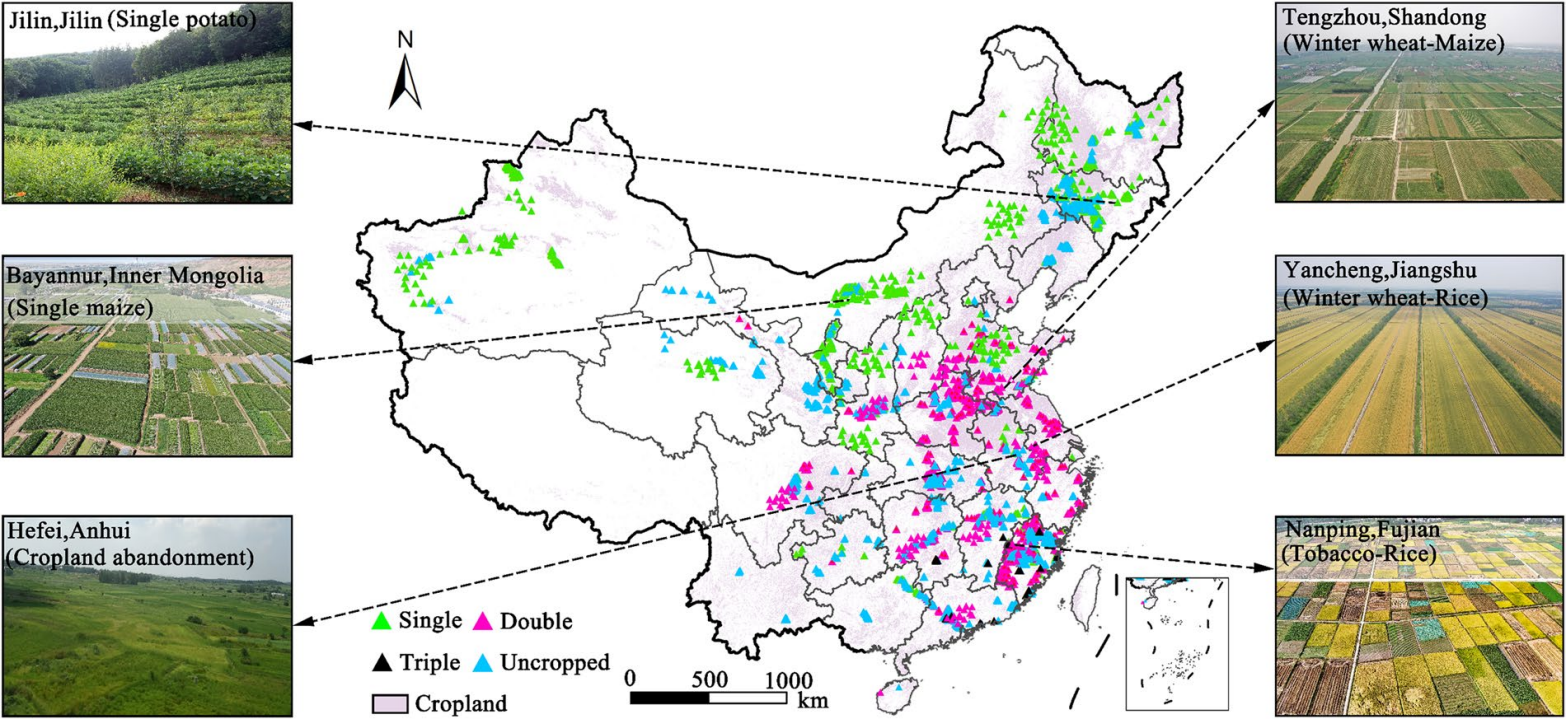
The distribution map of ground-truth reference sites in China.

Validation data included the ground-truth reference datasets and the agricultural census data. Ground truth reference datasets provided detailed information on cropland use intensity for each site. A field survey was conducted in major agricultural regions by our research group members and collaborators during the study period. Information on the location and land use types was recorded for each site. For the actively cropped fields, the crop types and cropping intensity were gathered. A total of 42,193 reference sites were collected primarily through field surveys and supplemented by a questionnaire (see locations in Fig. 2). Finally, we collected 6,759, 6,448, 15,645, 5,928, and 7,422 ground samples in 2018, 2019, 2020, 2021, and 2022, respectively. Specifically, there were 6,989, 17,509, 17,309, and 386 sites of uncropped, single, double, and triple cropping sites, respectively (Table S1). All reference sites were applied for accuracy assessment.

The officially reported agricultural census data delivered information on the total sown areas of crops at provincial and prefectural levels in China, which could be downloaded from the National Statistical Bureau of China (NSBC) (http://www.stats.gov.cn/english/). The cropland distribution dataset was derived based on the combinations of two 10-m global land cover datasets: the ESA_WorldCover and the FROM_GLC. The ESA_WorldCover and FROM_GLC datasets have achieved good accuracy, with 0.97 and 0.88, respectively. The union of cropland subsets from these two land cover datasets was applied as the mask of cropland in this study to eliminate possible omission errors of classification.

### Overview of the mapping approach for deriving cropland abandonment and cropping intensity

We applied robust algorithms and consistent procedures to estimate the cropland use intensity over conterminous China based on Google Earth Engine (GEE) using the S1 and S2 time series images. First, a cropland layer was generated based on two recent 10-m global land cover data products, the ESA_WorldCover and the FROM_GLC^29^. The cropland in northern China was derived from the ESA_WorldCover and pixels labeled as cropland in any of these above two land cover data products in southern China were applied as cropland layer to eliminate possible omission errors (here refer to 10-m UnionEF cropland dataset, Figure S1). Second, actively cropped fields were discriminated from fallow/abandoned land within the 10-m UnionEF Cropland by fusing Vegetation-Soil-Pigment indices and Synthetic-aperture radar (SAR) time-series images (VSPS)^44^. The cropped fields exhibited frequent and asynchronous changes among SAR and multiple spectral indices, attributable to cropping activities. These changes were discernible through three proposed knowledge-based temporal features. Finally, annual LUI datasets were produced through further Mapping cropping Intensity in actively cropped fields regarding improved characterized crop Life cycles based on combined considerations of vegetative and productive Stages (MILS)^28^. The 10-m LUI datasets in China were generated by automatic algorithms with no requirements for regional adjustments or intensive training samples.

### Discriminating actively cropped fields from fallow/abandoned land by the VSPS algorithm

When cropland is cultivated by field crops, it will be subject to farming activities such as plowing, sowing, irrigation, fertilization, and harvesting during the study year. Therefore, the actively cropped fields would experience tremendous changes in soil and vegetation cover together with Chlorophyll (Chl) concentration. This study applied a robust mapping framework for automatically identifying actively cropped fields in China at a national scale using the VSPS algorithm^44^. Knowledge-based features enhance the stability and robustness of the classifiers allowing large-scale applications over multiple years without recalibration^40^. Three knowledge-based temporal indicators were proposed to characterize the changes in land surfaces. The first temporal indicator, the VV-based temporal Variance (VVV), was developed based on the radar VV time series from S1 datasets. The second indicator, the Vegetation-Soil Differenced Temporal Variation (VSD), was designed to measure the extent of synchronicity between changes in vegetation and soil cover revealed by the corresponding spectral indices. The third indicator, the Vegetation-Pigment Differenced Temporal Variation (VPD), was developed to estimate the curvature of comparative changes between vegetation and pigment indices.

The actively cropped fields were expected to illustrate much higher values in the first indicator (VVV) or at least one from the latter two indicators (VSD, VPD). This is because any farming activities from actively cropped fields can be reflected from the varying VV backscatter (VVV) or large comparative variations among vegetation cover, soil cover, or pigment concentrations (VSD, VPD). When cropland is lying fallow or abandoned, the land surfaces remain more stable compared to actively cropped fields, which could be revealed by lower values in these three proposed temporal indicators. A straightforward decision model was developed to discriminate actively cropped fields from fallow/abandoned land. The thresholds in the decision rule were determined based on the histogram of randomly selected reference sites^44^. These thresholds were applied nationally without additional regional or yearly adjustments. The VSPS algorithm demonstrated strong performance in China in 2020^44^ and was utilized here to generate distribution maps of actively cropped fields over the past six years.

### Mapping cropping intensity on actively cropped fields using the MILS algorithm

Information on cropping intensity is generally derived based on VI waves^41^. VI-waves-based approaches have been widely applied to derive cropping intensity datasets from national to global scales, which were primarily focused on detecting valid VI peaks^9^. Optical time series images such as the 500 m Moderate Resolution Imaging Spectroradiometer (MODIS) surface reflectance products and 30 m Landsat images were commonly applied for this purpose^11,45,46^. However, it is challenging to generate 10-m national-scale CI data products in China with good quality by merely applying the VI waves. These challenges included the frequently observed multiple VI waves within one cropping cycle (i. e. the winter wheat), the complexity of cropping systems, and the deficiency of finer-resolution time series datasets with adequate frequency and spectral information^47^. The second cropping cycle of double rice is commonly underestimated due to rapid late rice transplanting after early rice harvesting^41^. These few 10-m CI mapping efforts in China were constrained in some regions or specific years^22,48^.

We coped with these above challenges by proposing a novel and robust approach for Mapping cropping Intensity with improved quantification of crop Life cycles with combined considerations of vegetative and productive Stages (MILS) using the Sentinel-1 (S1) SAR and Sentinel-2 (S2) MSI time series images. We detected the numbers of valid coupled patterns of vegetation and brownness indices and then further improved by incorporating Sentinel-1 VH data to reduce the omission errors of double rice^28^. Cropping cycles can be described by the coupled dynamic patterns of vegetation and brownness indices. There are two kinds of VI-BRI coupled patterns: one is the coherent X-shaped pattern, and another is the double-nested pattern. The coherent X-shaped pattern is a typical VI-BRI coupled pattern observed from many crops including paddy rice, maize, wheat, soybean, peanut, and cotton. The coherent X-shaped pattern is characterized by the upside X-shaped feature (VI rise-BRI drop) followed by the downside X-shaped feature (VI drop-BRI rise), which reflected the VI-rising & BIR-dropping during the vegetative stage and VI-dropping & BRI-rising during the reproductive stage.

Besides the commonly examined coherent X-shaped pattern, the cropping cycles of a few economic crops can be characterized by the double nested pattern of VI and BRI. For example, the growth cycles of oilseed and tobacco can be depicted by the Co-up feature (VI rise-BRI rise) during the vegetative stage followed by the Co-down feature (VI drop-BRI drop) during the reproductive stage. The co-occurrence of VI-BRI peaks in oilseed and tobacco is attributed to the long periods of yellow flowers of oilseeds and large leaves abundant in the carotenoid concentration of tobacco crop, which are different from most crops showing high BRI values during heading stages. The double nested pattern was incorporated to cope with the complexity of the cropping systems. Although there might be multiple VI waves during one growing cycle, there is only one valid VI-BRI coupled pattern examined, which could be either the coherent X-shaped pattern or the double nested pattern of VI and BRI. The frequency of all valid VI-BRI coupled patterns was computed through a per-pixel strategy for each year and the spatiotemporal continuous CI datasets were generated for the study area. All the procedures were implemented on the GEE platform.

## Data Records

**Fig. 3:**
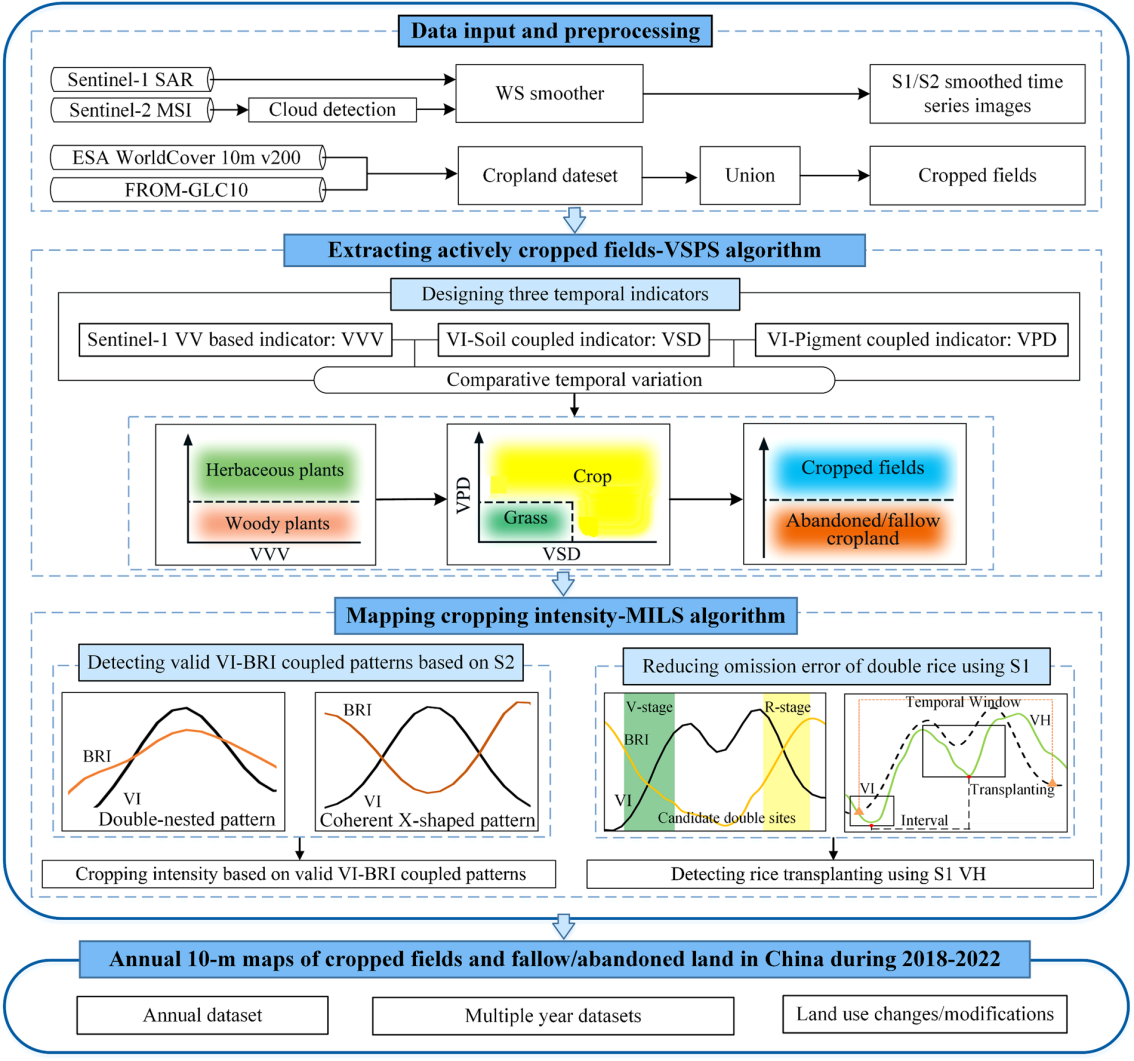
The workflow of the cropland uses an intensity mapping algorithm.

There were three groups of national-scale datasets on cropland use intensity over conterminous China during the study period (2018–2023). The first group of datasets was the cropland use intensity dataset for each year. Six 10-m national scale cropland use intensity maps (ChinaCUI10m) were provided for China during the study period. The second group of datasets was the newly derived 10-m cropland dataset (ChinaCropland10m) based on historical cropping activities. The historical cropland use intensity during the study period was summarized and the ChinaCropland10m was produced by discarding pixels with values of zero (cropland should be actively cropped at least once during the past six years) (Fig. 2). The third group of datasets was the degree of intensification revealed by averaged cropland use intensity (ChinaMeanCI10m) based on historical CUI datasets in China during the study period. The datasets are available in the figshare repository in a Geotiff format^49^. The dataset is provided in ESPG: 4326 (WGS_1984) spatial reference system. For the first group data (ChinaCUI10m), there are four values: {0: fallow; 1: single cropping; 2: double cropping; 3: triple cropping}. For the third group data (ChinaCropland10m), there are thirteen values ranging within [0,3], with greater values indicating higher cropland use intensity Fig. 3.

## Technical Validation

### Site-level comparison with ground-truth data

We validated our produced cropland use intensity maps (ChinaCropland10m) using all the ground survey datasets during its corresponding year. The overall accuracy (OA), user accuracy (UA), producer accuracy (PA), and F1-score (F1) were computed based on the reference data. Accuracy assessment results based on reference sites were provided in Table 1. An overall accuracy of 90.88% was achieved, with the kappa index of 0.8704. The F1 scores of these four types of cropland use intensity varied from 0.87 to 0.92. The cropping intensity of single and double cropping was accurately labeled with F1 scores of above 0.90 (0.91–0.92).

**Table 1:** Accuracy assessment using ground reference sites.

S1/2 estimates	Reference data						
	Total	Fallow	Single	Double	Triple	Producer accuracy (%)	F1
Fallow	6989	6123	652	211	3	87.61	0.88
Single	20518	605	16478	425	1	94.11	0.91
Double	17309	229	1634	15444	2	89.23	0.92
Triple	386	13	23	49	301	77.98	0.87
User accuracy (%)		87.85	87.71	95.75	98.05		
Overall accuracy (%)	90.88						
Kappa	0.8555						

Classification of fallow/abandoned cropland obtained considerably lower accuracy than single or double cropping, with an F1 score of 0.88. The PA and UA of single cropping and double cropping were greater than 0.9. Triple cropping had much higher UA than PA, which suggested that triple cropping was underestimated (omission errors). Among these 386 sites of triple cropping, 85 sites were not correctly labeled. Double cropping also illustrated higher UA than PA, which suggested that multiple cropping patterns were associated with more omission errors. It is because more valid observations are required to fully characterize two or three crop growing cycles, which is generally constrained by the lower data availability in southern China^28^.

The classification accuracy is influenced by the data availability of Sentinel-2 MSI images (Fig. S2). The overall accuracy was above 0.92 with good data availability (>50% valid observations). The overall accuracy sharply decreased when the percentages of valid observations were less than 25%. Luckily, there were only around 10% of cropland in China illustrated with less than 30% of valid observations in Sentinel-2 MSI data. Pixels with serious cloud contaminations were mainly located in southwest China. Therefore, a majority of cropland obtained good accuracy in China with the robust mapping algorithms.

### Province-level comparison with agricultural census data

**Fig. 4:**
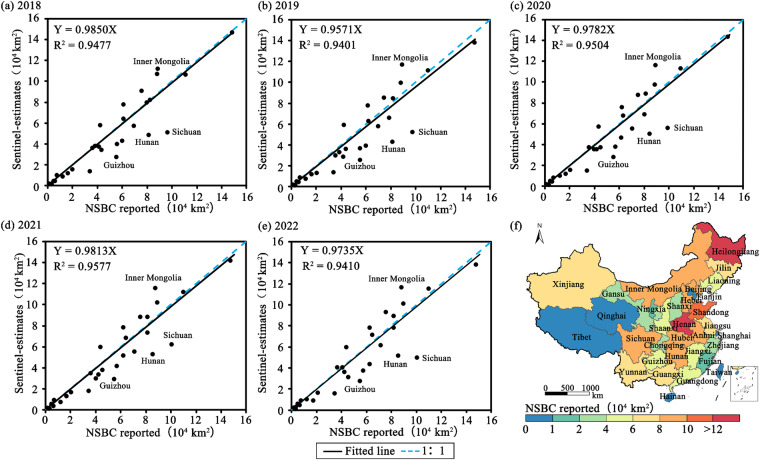
Comparisons between NSBC reports and S1/2 estimates (**a**–**e**) and map of officially reported sown area in 2019 (**f**).

The total sown areas were estimated from the cropland intensity dataset and compared with the officially reported agricultural census data at the provincial level for each year (Fig. 4). The estimated sown areas from the ChinaGUI10m datasets were highly correlated with those from the agricultural census data, with R2 of no less than 0.94 for these past six years. Most provinces obtained consistent results with the statistical data during the study period. Overestimations were illustrated in several provinces of northern China such as Inner Mongolia. The total sown area in some southern provinces (i.e. Hunan, and Sichuan provinces) had lower sown areas than the agricultural census data, which might be associated with the mixed-pixel problem and possible overestimation of agricultural census data in the yearbook. Cropland in southern China is fragmented and these very small-sized cropped fields in mountainous and hilly regions are subject to uncertainties due to the mixed pixel problem. The local governments and farmers tended to report more areas cultivated by crops to benefit from agricultural subsidies^50^.

### Comparisons with GCI30 data product

**Fig. 5:**
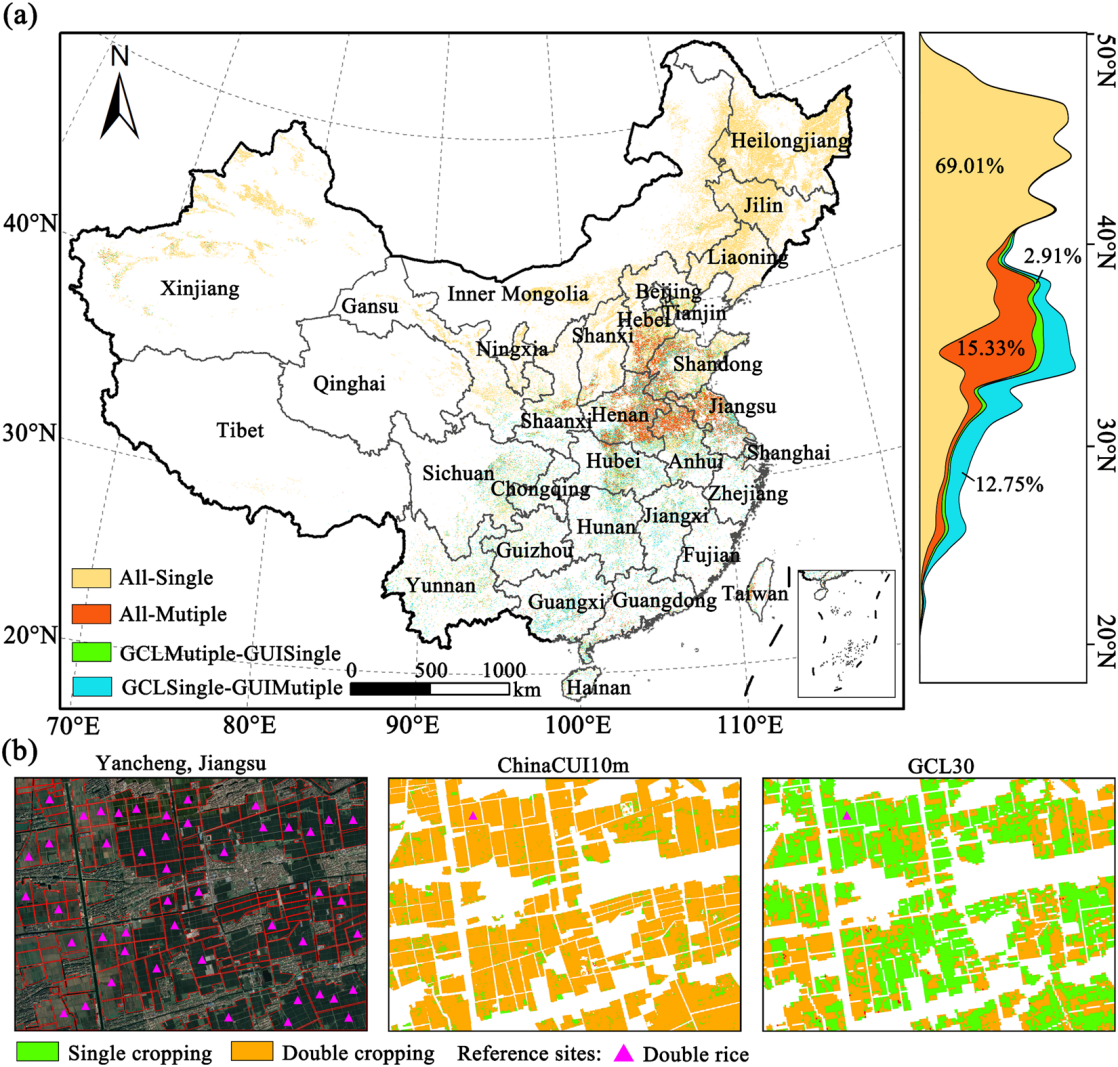
The map of consistency and discrepancies between ChinaGUI10m and GCI30 dataset in China (s) and some snapshots (b).

The comparison between the 10 m cropland use intensity map and the 30 m global cropping intensity data products (GCI30)^51^ revealed a consistent spatial pattern of single and multiple cropping across China. Single cropping predominated in northern China, while multiple cropping was prevalent in the HuangHuaiHai plain. Approximately 69.01% and 15.33% of cropland were consistently identified as single or multiple cropping, respectively, across both CI data products (seen in Fig. 5). Discrepancies were observed in 15.67% of cropland. Notably, 12.76% of cropland was labeled as multiple cropping exclusively in the ChinaGUI10m dataset, primarily concentrated in southern China.

The underestimation of multiple cropping in the GCI30 data products can be attributed to the omission error of double rice and the absence of cropping cycles due to frequent cloud contamination or mismatched vegetation index peaks during both early and late growing seasons^28,51^. Consequently, systematic underestimations were evident in the GCI30 data product, particularly in regions of southern China characterized by cloudy weather and intricate cropping systems.

## Usage Notes

**Fig. 6:**
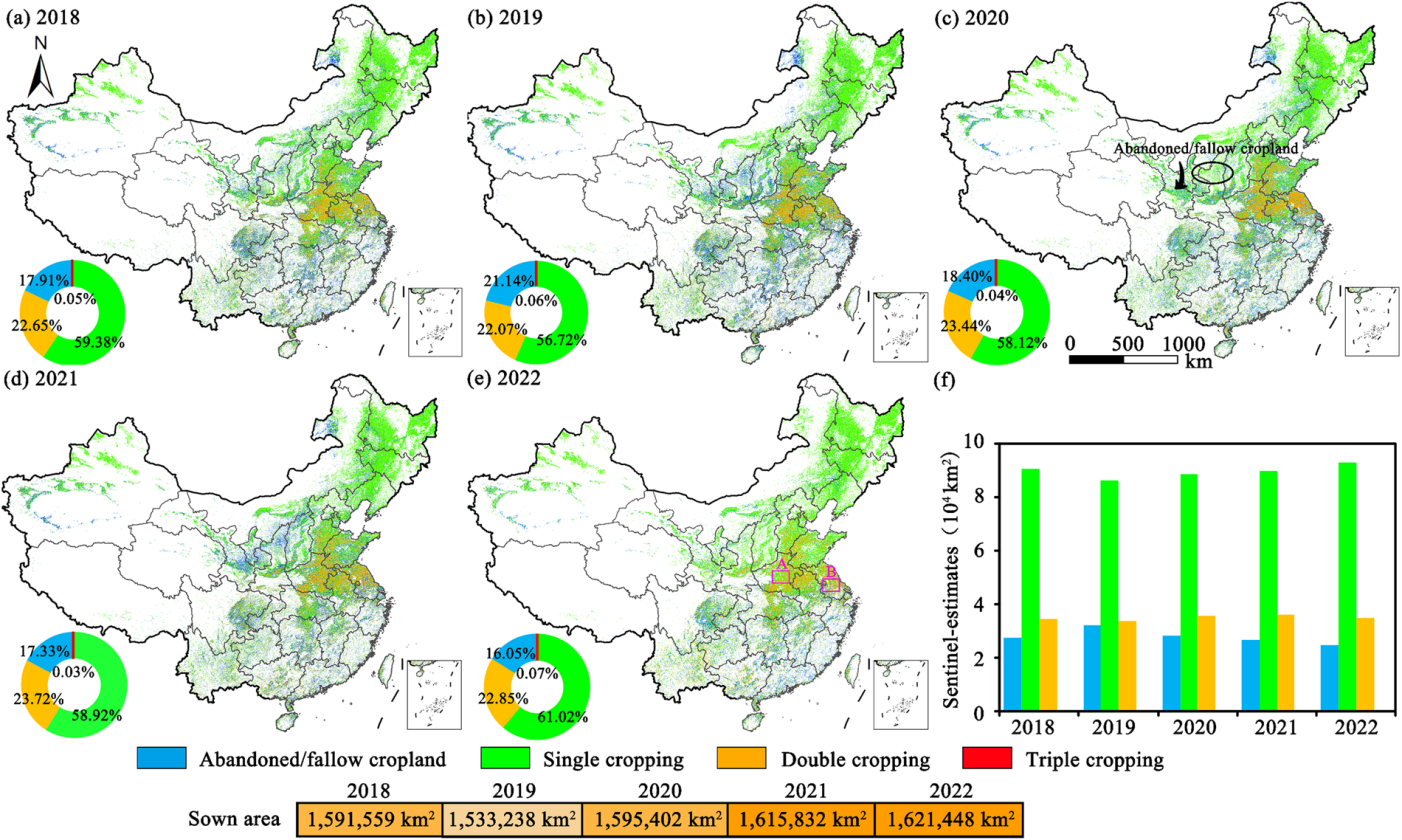
Annual maps of cropland use intensity (**a**–**e**) and total areas of different cropping intensity in China (**f**).

China ranks at the top in the global food bowl of a series of crops such as rice and wheat. Spatiotemporal explicit data on cropland use intensity in China in recent years is vital for ensuring national or global food security. This study provided the 10-m national-scale distribution maps of cropland use intensity during the study period (Fig. 6). The distribution maps of actively cropped fields and cropping intensity are the foundation for operational agricultural monitoring systems such as crop identification, crop growth, and crop yield estimations. The ChinaCUI10m datasets can be exploited to support a wide range of studies towards a series of development goals such as stabilizing agricultural production enhancing farmer’s incomes, and reducing carbon emissions and agricultural disasters^17,52^.

**Fig. 7:**
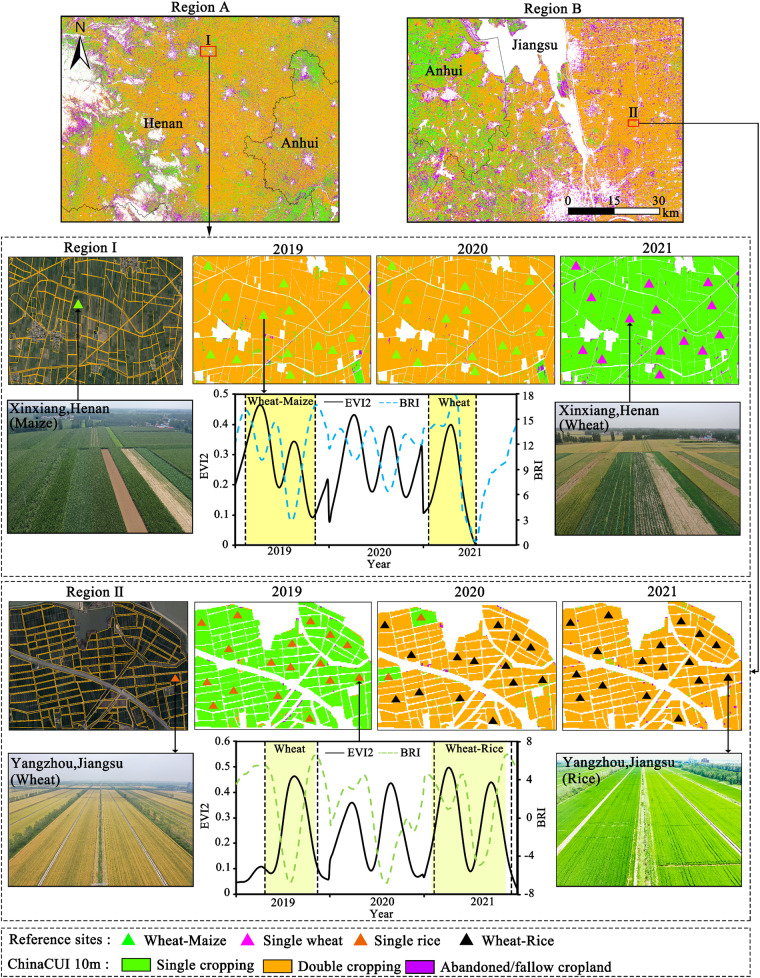
Some snapshots of cropland use intensity in HuangHuaiHai and Yangtze River plains in China. Notes: see the locations of regions A and B in Fig. 5.

National-scale annual agricultural datasets with finer resolutions (10 m) provide quantitative information on the changes of actively cropped fields and switches between single or multiple cropping in the cropping systems, which is vital to evaluate the performances of recent agricultural policies such as strictly prohibiting cropland abandonment, preventing non-grain productions (maintaining grain self-sufficiency), and promoting rural revitalization^53,54^. We provided the first spatiotemporal continuous national-scale cropland use intensity datasets which provided detailed descriptions and changes on fallow, single, and multiple cropping in China during 2018–2023 (some snapshots are shown in Fig. 7). Results showed that the total sown areas in China were relatively low in 2019, which continuously enhanced after 2019 (Fig. 4). The continuously enhanced sown areas since 2019 were mainly accounted for by the increase of single cropping patterns. The spatiotemporal continuous datasets enable us to track the long-term changes in the agricultural systems, which could be updated to 2023 or the coming years to reflect near real-time changes in cropland and agricultural practices.

Sustained productivity of stable cropland is crucial for ensuring global food security^55^. Sustainable intensification in cropping systems is essential to meet the increasing food demand of the global population^56^. Achieving sustainability in land use systems is challenged by a series of issues such as abrupt changes and complex driving forces^57^. Land change science will continue to play a key role in addressing a series of global socio and environmental challenges^58^. We provided the first national-scale 10-m accurate cropland data by tracking the historical cultivation activities in the past six years. The newly updated 10-m cropland and averaged cropland intensity maps can be used to support related studies toward targeted sustainable intensification in agricultural systems^59,60^.

### Supplementary information

Supplement

## Data Availability

The cropland use intensity mapping algorithms were implemented in GEE. The map processing codes and produced cropland use intensity datasets were available at 10.6084/m9.figshare.24603234. Datasets of cropland use intensity could be automatically generated in China or other countries/regions based on the publicly accessible S1/S2 time series images using the shared processing code, which does not require training samples or human interactions.
