# Supplementary material for: National-scale 10-m maps of cropland use intensity in China during 2018–2023
Source: Sci Data. 2024 Jun 26;11:691. doi: 10.1038/s41597-024-03456-0 (PMC11208577; doi:10.1038/s41597-024-03456-0)
Supplement: Supplementary file 1 — Supplement [file 41597_2024_3456_MOESM1_ESM.docx]

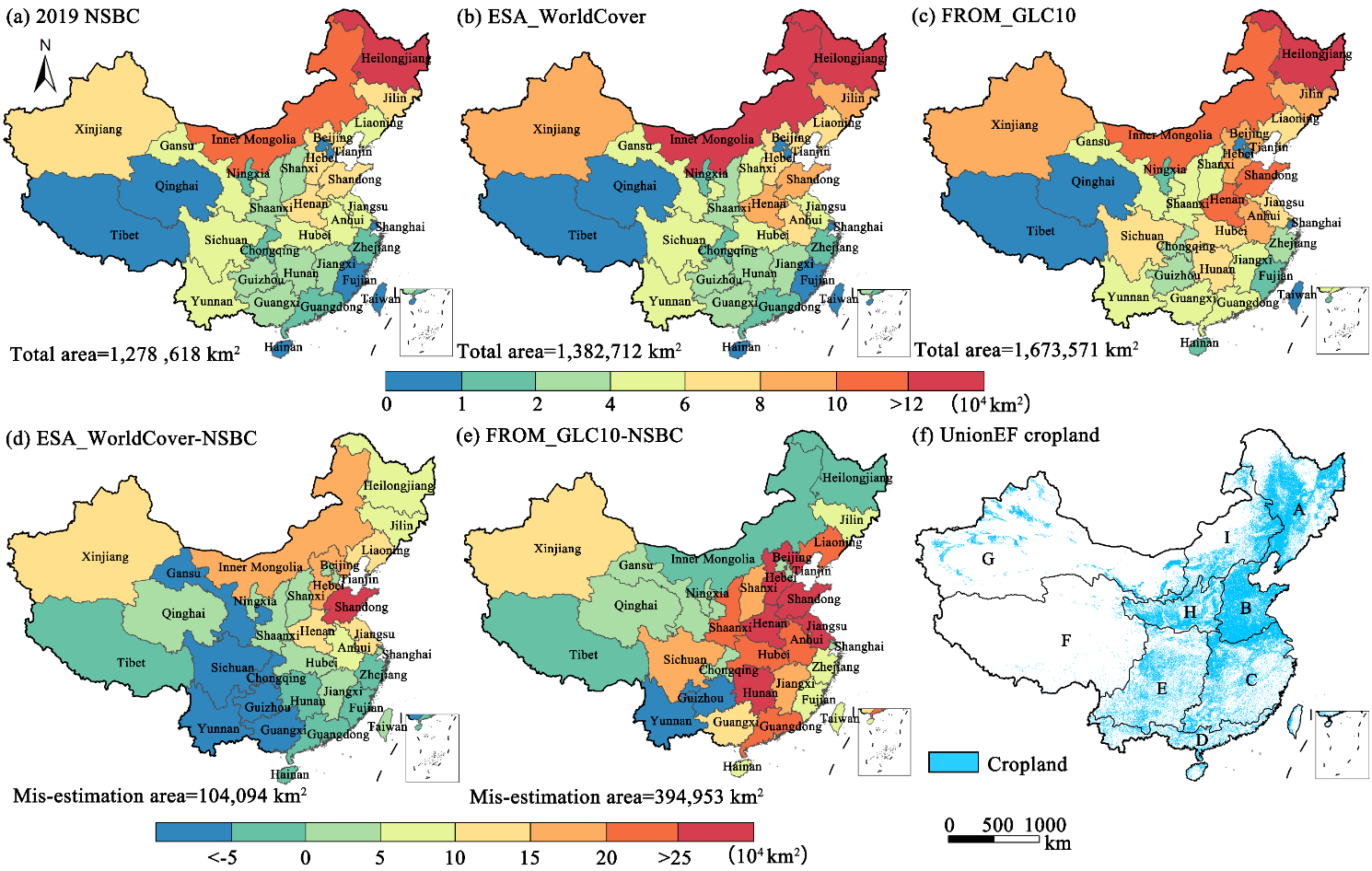


Figure S1. Maps of (a) NSBC officially reported cropland data, (b) ESA-WorldCover, (c) FROM-GLC10, (d, e) differences between NSBC-reported and these two global land cover datasets, and (f) their unions (UnionEF).

Notes: Northeast China (A), Huang-Huai-Hai Plain (North China Plain, B), Middle and lower reaches of the Yangtze River Plain (C), South China (D), Southwest China (E), Qinghai-Tibet (F), Gan-Xin (G), Loess Plateau (H), and Inner Mongolia and along the Great Wall (I).


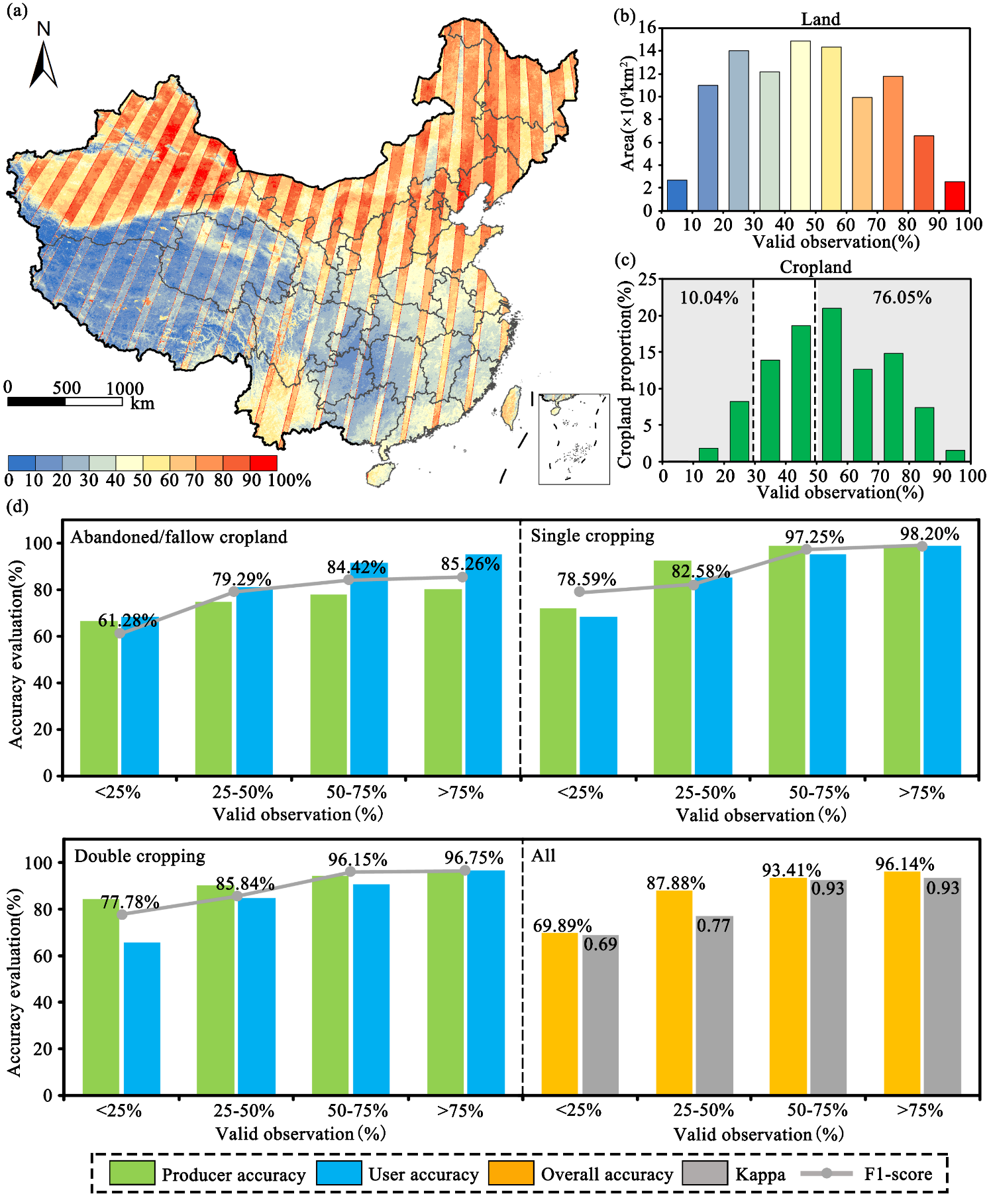


Figure S2. Maps of percentages of valid observations in Sentinel-2 MSI data in China in 2020 (a) and changes of classification accuracy with the percentages of valid observation (b).

Table S1. Information on reference sites

|  | 2018 | 2019 | 2020 | 2021 | 2022 | Total |
| --- | --- | --- | --- | --- | --- | --- |
| Fallow | 1419 | 1702 | 993 | 1314 | 1561 | 6989 |
| Single | 3376 | 2557 | 7641 | 1778 | 2157 | 17509 |
| Double | 1883 | 2134 | 6844 | 2800 | 3648 | 17309 |
| Triple | 81 | 46 | 167 | 36 | 56 | 386 |
| Total | 6759 | 6448 | 15645 | 5928 | 7422 | 42193 |
